# The amplitude of low-frequency fluctuation characteristics in depressed adolescents with suicide attempts: a resting-state fMRI study

**DOI:** 10.3389/fpsyt.2023.1228260

**Published:** 2023-07-28

**Authors:** Changchun Hu, Wenhao Jiang, Jie Huang, Jian Lin, Jialing Huang, Mei Wang, Jian Xie, Yonggui Yuan

**Affiliations:** ^1^Department of Clinical Psychology, Affiliated Hangzhou First People’s Hospital, Zhejiang University School of Medicine, Hangzhou, China; ^2^Department of Psychosomatics and Psychiatry, Zhong Da Hospital, School of Medicine, Southeast University, Nanjing, China; ^3^Affiliated Hangzhou First People’s Hospital, Zhejiang University School of Medicine, Hangzhou, China

**Keywords:** adolescent, depression, amplitude of low-frequency fluctuation, suicide attempts, emotion dysregulation

## Abstract

**Background:**

The amplitude of low-frequency fluctuation (ALFF) is a measure of spontaneous brain activity derived from resting-state functional magnetic resonance imaging (rs-fMRI). Previous research has suggested that abnormal ALFF values may be associated with major depressive disorder (MDD) and suicide attempts in adolescents. In this study, our aim was to investigate the differences in ALFF values between adolescent MDD patients with and without a history of suicide attempts, and to explore the potential utility of ALFF as a neuroimaging biomarker for aiding in the diagnosis and prediction of suicide attempts in this population.

**Methods:**

The study included 34 adolescent depression patients with suicide attempts (SU group), 43 depression patients without suicide attempts (NSU group), and 36 healthy controls (HC group). Depression was diagnosed using a threshold score greater than 17 on the Hamilton Depression Rating Scale (HDRS). The rs-fMRI was employed to calculate zALFF values and compare differences among the groups. Associations between zALFF values in specific brain regions and clinical variables such as emotion regulation difficulties were explored using Pearson partial correlation analysis. Receiver-Operating Characteristics (ROC) analysis assessed the ability of mean zALFF values to differentiate between SU and NSU groups.

**Results:**

Significant differences in zALFF values were observed in the left and right inferior temporal gyrus (l-ITG, r-ITG) and right fusiform gyrus (r-FG) among the three groups (GRF corrected). Both SU and NSU groups exhibited increased zALFF values in the inferior temporal gyrus compared to the HC group. Furthermore, the SU group showed significantly higher zALFF values in the l-ITG and r-FG compared to both the NSU group and the HC group. Partial correlation analysis revealed a negative correlation between zALFF values in the left superior and middle frontal gyrus (l-SFG, l-MFG) and the degree of emotional dysregulation in the SU group (*R* = −0.496, *p* = 0.003; *R* = −0.484, *p* = 0.005). Combining zALFF values from the l-ITG and r-FG achieved successful discrimination between depressed adolescents with and without suicide attempts (AUC = 0.855) with high sensitivity (86%) and specificity (71%).

**Conclusion:**

Depressed adolescents with suicidal behavior exhibit unique neural activity patterns in the inferior temporal gyrus and fusiform gyrus. These findings highlight the potential utility of these specific brain regions as biomarkers for identifying suicide risk in depressed adolescents. Furthermore, associations between emotion dysregulation and activity in their frontal gyrus regions were observed. These findings provide preliminary yet pertinent insights into the pathophysiology of suicide in depressed adolescents.

## 1. Introduction

Suicide is a major public health concern among adolescents, with depression being a significant risk factor (1). Depression is prevalent in this population and has a substantial impact on their overall well-being and functioning (1). Data shows that the lifetime prevalence of major depressive disorder among individuals aged 13–18 is 11.0% (2), and the incidence of depression is rising more rapidly among adolescents than adults (1, 3). According to the World Health Organization (WHO), suicide is the second leading cause of death globally for those 15–29 year olds (4). Adolescence represents a critical period for brain maturation and socio-psychological development. Changes in brain development during this time may increase the risk of suicide while also providing an opportunity to better understand this risk (5). As such, it is essential to investigate the neurobiological mechanisms underlying suicidal behavior in depressed youths to develop effective prevention and intervention strategies.

The amplitude of low-frequency fluctuation (ALFF) measures the spontaneous neural activity in resting-state functional magnetic resonance imaging (6). Several studies have reported abnormal ALFF values in various brain regions of depressed adolescents with suicide attempts compared to healthy controls or depressed adolescents without suicide attempts. These regions include the medial superior frontal gyrus (mSFG), the precuneus, the middle and inferior frontal gyri, the middle and superior temporal gyri, the occipital cortex, the anterior cingulate cortex (ACC), the medial prefrontal cortex (mPFC), the hippocampus, and the thalamus (7, 8). The abnormal ALFF values in these regions may reflect impaired functions such as emotion regulation, self-referential processing, cognitive control, memory, and salience detection (9). However, these findings are not consistent and poorly replicable across studies and may be influenced by factors such as sample size, age range, and comorbidity. Therefore, more rigorous and standardized studies are needed to clarify the ALFF differences between depressed adolescents with and without suicide attempts and to explore the underlying neural mechanisms of suicidal behavior.

Emotion dysregulation refers to the inability to identify, understand, and manage one’s own emotions in an adaptive way (10, 11). Several studies have suggested that emotion dysregulation plays a significant role in developing and maintaining depression and suicide-related behaviors in adolescents (12–14), which confers prospective vulnerability to psychopathology in adulthood. However, existing studies have predominantly focused on examining emotion dysregulation as a broad risk factor for suicidal behavior, overlooking the potential neurobiological underpinnings of suicide, especially alterations in brain functioning as detected through imaging techniques (15, 16). According to the developmental theory of emotion dysregulation, neurodevelopment of the prefrontal cortex lags several years behind neurodevelopment of subcortical structures (17). Recent research has demonstrated that in adolescents with internalizing disorders, emotion dysregulation is significantly associated with impaired neurodevelopment of the prefrontal cortex and reduced efficiency of subcortical–cortical connections (18, 19). Thus, we hypothesize that there may be a relationship between the degree of emotion dysregulation and prefrontal cortex activation in depressed adolescents with a history of suicide attempts.

In light of the exploratory nature of this study, our primary objective is to investigate the relationship between the amplitude of ALFF abnormalities and suicide attempts in depressed adolescents using rs-fMRI whole-brain analysis. Specifically, we aim to (1): Determine differences in ALFF values among three groups: depressed adolescents with suicide attempts (SU group), depressed adolescents without suicide attempts (NSU group), and healthy controls (HC group) (2). Although our primary focus was on the prefrontal cortex, for the sake of completeness, we also performed whole-brain analyses to explore the association between changes in ALFF values in other regions and Emotion dysregulation related to depression and suicide risk (3). Assess the discriminative accuracy of group differences in mean ALFF values across brain regions in distinguishing between depressed adolescents with and without suicide attempts. The significance of this study lies in its potential to advance our understanding of the neurobiological mechanisms underlying suicidal behavior in depressed adolescents. By conducting a whole-brain analysis of ALFF characteristics, we can identify specific brain regions that may be implicated in the pathophysiology of suicide attempts.

## 2. Materials and methods

### 2.1. Subjects

A total of 77 adolescent patients with depression and 36 healthy volunteers (HC control group) were recruited from Hangzhou First People’s Hospital between January 2020 and December 2021. Gender and age were meticulously matched between the patient and control groups. All participants were of Han Chinese ethnicity, right-handed, and aged between 12 and 21. Adolescent patients with depression are typically inpatients in a psychiatric department of a hospital, seeking help and treatment for their condition.

Comprehensive diagnostic assessments were administered by trained clinicians, with separate interviews conducted with both adolescents and their parents. Participants under 18 years old underwent assessment using the Kiddie Schedule for Affective Disorders and Schizophrenia-Present and Lifetime Version (K-SADS-PL), while those aged 18 years or older were assessed using the Structured Clinical Interview for Statistical Manual of Mental Disorders, Fourth Edition (DSM-IV) Axis I Disorders (SCID). Major depressive disorder (MDD) was diagnosed using a threshold score greater than 17 on the Hamilton Depression Rating Scale (HDRS). Exclusion criteria encompassed the presence of other psychiatric axis-I comorbidities, neurological illnesses, a history of substance or alcohol abuse/dependence at any time, electroconvulsive therapy, head trauma resulting in loss of consciousness, and other clinically relevant abnormalities identified through medical history or laboratory examination.

The control group comprised 36 healthy individuals matched with the patient group regarding gender, age, and education level. The Structured Clinical Interview for DSM-IV, non-patient edition (SCID-NP) was administered to control participants to ensure their absence of past or current DSM-IV Axis I diagnoses. Individuals with a history of substance or alcohol abuse/dependence, electroconvulsive therapy, head trauma resulting in loss of consciousness, or other clinically relevant abnormalities identified through medical history or laboratory tests were excluded. Ethical approval for the study was obtained from the Medical Ethics Committee of Hangzhou First People’s Hospital (IRB: 2020-K008-01). All participants or their legal guardians received detailed information about the study’s objectives and procedures, and they provided informed consent before participation.

### 2.2. Clinical assessment

According to the definition of “suicide attempt” in the Columbia Suicide Assessment Classification System, the history of suicide attempts includes the number of previous suicide attempts and the time since the last suicide attempt (20). The patients were divided into the depression with suicide attempt group (SU) and depression without suicide attempts group (NSU) by confirming through clinical records and interviews with the subjects and their families. On the day of the MRI scan, the severity of depression was measured by HDRS; Beck Scale assessed the severity of suicidal ideation for Suicide Ideation (SSI); and the degree of emotional dysregulation was evaluated by Difficulty in Emotional Regulation Scale (DERS) (21). The history of psychotropic drug treatment was obtained from their clinical records. Among these patients, 9 suicide attempters and 11 non-suicide attempters were taking medication for depression. Medication included antidepressant medication and augmentation (see Table 1).

**Table 1:** Comparison of demographic and clinical data of the three groups of subjects.

Variables	SU (*n* = 34)	NSU (*n* = 43)	HC (*n* = 36)	*F/χ^2^/t*	*p*-value
Age (years)	16.35 ± 2.76 (12–21)	16.44 ± 2.28 (12–21)	17.08 ± 2.76 (12–21)	0.859^a^	0.426
Gender(M/F))	11/23	15/28	12/24	0.057 ^b^	0.972
Education (years)	9.61 ± 2.08	10.03 ± 2.54	10.81 ± 3.20	1.35	0.264
HAMD	25.62 ± 6.53	23.85 ± 5.40	—	1.30 ^c^	0.197
DERS(total scores)	130.18 ± 21.93	116.56 ± 27.30	—	2.35 ^c^	0.022
SSI	23.25 ± 7.75	14.60 ± 9.61	—	4.53 ^c^	<0.001
Medication(yes/no)	9/26	11/25	—	0.008 ^b^	0.93
Antidepressants	9	11	—		
Antipsychotics	4	2	—		
Mood stabilizer	2	0	—		

SU: Suicide Attempts; NSU: Non-Suicide Attempts; HC: healthy controls; DERS: Difficulty in Emotional Regulation Scale; SSI: Beck Scale for Suicide Ideation; HAMD: Hamilton Depression Scale; a indicates *F* for one-way ANOVA; b indicates *χ*^2^ for chi-square test; c indicates *t* for the two-sample *t*-test.

### 2.3. Acquisition of rs-fMRI data

In this study, we acquired data using a 3 T MAGETOM Verio scanner (Erlangen, Germany) with a 32-channel phased-array head coil. Before scanning, the technician explained the procedure to the subjects, including the possible noise and claustrophobic environment during the test, and instructed them to stay awake, close their eyes, and keep their heads still during the scanning process. The head was fixed for braking, and earphones and earplugs were worn to reduce noise. The data included 3D-T1 structural imaging and resting-state functional magnetic resonance imaging (rs-fMRI), which were part of a larger assessment that also included diffusion tensor imaging (DTI). However, we did not perform task-related MRI. The total scanning time for each subject was approximately 40 min, with the rs-fMRI scan taking 8 min and 06 s and the 3D-T1 structural imaging scan taking 4 min and 18 s. For 3D-T1 structural imaging, we used a TR of 1900 ms, TE of 2.52 ms, slice thickness/gap of 1 mm/0 mm, flip angle of 9°, and a field of view of 256 × 256 mm with a matrix size of 256 × 256. For rs-fMRI data acquisition, we used a TR of 2000 ms, TE of 30 ms, slice thickness/gap of 3.2 mm/0 mm, a total of 47 slices, flip angle of 90°, a field of view of 220 mm × 220 mm, matrix size of 64 × 64, and collected a total of 240 time points.

### 2.4. Preprocessing of rs-fMRI data

We used the Matlab R2018b platform (The MathWorks, Inc., Natick, Mass., http://www.mathworks.com/products/matlab) and the DPABI_V4.0 toolbox (Data Processing Assistant for Resting-State Functional MRI) (22) for preprocessing of magnetic resonance images. This toolbox is based on REST (Resting-State Functional MRI, http://www.restfmri.net) and SPM12. Before preprocessing, we discarded the first 10 time points of the fMRI data to avoid the instability caused by initial magnetic field fluctuations. The remaining 230 rS-fMRI images were temporally and spatially corrected with the reference plane being the 36th slice. Subjects with maximum displacements exceeding 1.5 mm or 1.5 degrees of motion were excluded. The 3D-T1 weighted images were segmented and normalized to 3 mm isotropic MNI standard space, and the RS-fMRI data were registered to the corresponding high-resolution T1-weighted anatomical image. Finally, the co-registered fMRI images were normalized to the standard MNI template, resampled to a voxel size of 3 mm × 3 mm × 3 mm, and spatially smoothed using a Gaussian kernel with a full width at half maximum of 6 mm.

### 2.5. ALFF analysis

We utilized DPABI software to calculate voxel-wise ALFF values for each subject. The preprocessing steps involved detrending and band-pass filtering (0.01–0.08 Hz) of the fMRI data (22). Subsequently, a fast Fourier transform was employed to convert the time series of each voxel into the frequency domain, resulting in a power spectrum. To derive the ALFF value, we performed a square root transformation on each frequency of the power spectrum and calculated the average square root within the 0.01–0.08 Hz frequency range. To enhance the normality and reliability of the data, we applied a Z-score transformation to normalize the ALFF values of each voxel, accounting for variations in absolute signal intensity (23). The resulting zALFF images were utilized for subsequent statistical analyses.

### 2.6. Statistical analysis

For the analysis of demographic and clinical data, we utilized SPSS 26.0 software (SPSS, Inc., Chicago, Illinois). One-way analysis of variance (ANOVA) or t-tests were employed to compare count data, while chi-square tests were used to examine differences in measurement data.

Voxel-based comparisons of zALFF maps among the three groups were conducted using a one-way ANOVA design model in DPABI software, controlling for age and gender. Gaussian Random Field (GRF) correction was then applied to address multiple comparisons, with a voxel level threshold of *p* < 0.001 and a cluster level threshold of *p* < 0.05. This correction method enabled the identification of statistically significant differences, which were then considered regions of interest (ROIs). *Post hoc* two-sample t-tests were subsequently performed within these ROIs to assess between-group differences in zALFF values, also employing GRF correction for multiple comparisons.

Pearson correlation analysis was performed separately between the zALFF maps of the whole brain and the clinical characteristics (HDRS scores, DERS scores, number of suicide attempts and current SSI) of patients in the SU and NSU groups, controlling for age and gender using partial correlation analysis. GRF correction was applied for multiple comparisons, with a corrected voxel level of *p* < 0.001 and a cluster level of *p* < 0.05.

To assess the discriminative ability to mean zALFF values from intergroup differential brain regions between SU and NSU patients, receiver operating characteristic (ROC) analyses were conducted to determine sensitivity and specificity. Cutoff scores that yielded optimal balance between sensitivity and specificity (Youden Index) were used to calculate sample-based sensitivity/specificity proportions. Additionally, mean zALFF values from regions showing significant effects were incorporated to improve classification performance between groups. These values were treated as independent variables in a binary logistic regression equation, with the diagnostic group as the dependent variable. The predicted probability output from the regression model was used as the test variable, assuming a nonparametric distribution, with the diagnostic group as the state variable.

## 3. Results

### 3.1. Demographic data and clinical characteristics

The three groups had no statistical differences in gender, age, and years of education, but patients of the SU group manifested significantly higher total DERS scores(*t* = 2.35, *p* = 0.022) and SSI scores(*t* = 4.53, *p* < 0.001) compared with those of the NSU groups. No significant differences in HAMD scores and Medication were found between SU and NSU groups (see Table 1). Out of the patients of the SU group, 24 individuals (70.6%) had a recent suicide attempt within the past month. The average number of previous suicide attempts was 1.68 ± 0.98 times. The maximum number of suicide attempts observed was 5, with the majority of adolescents (29 participants, 85.3%) having made 1–2 suicide attempts.

### 3.2. ALFF differences between The three groups

**Figure 1:**
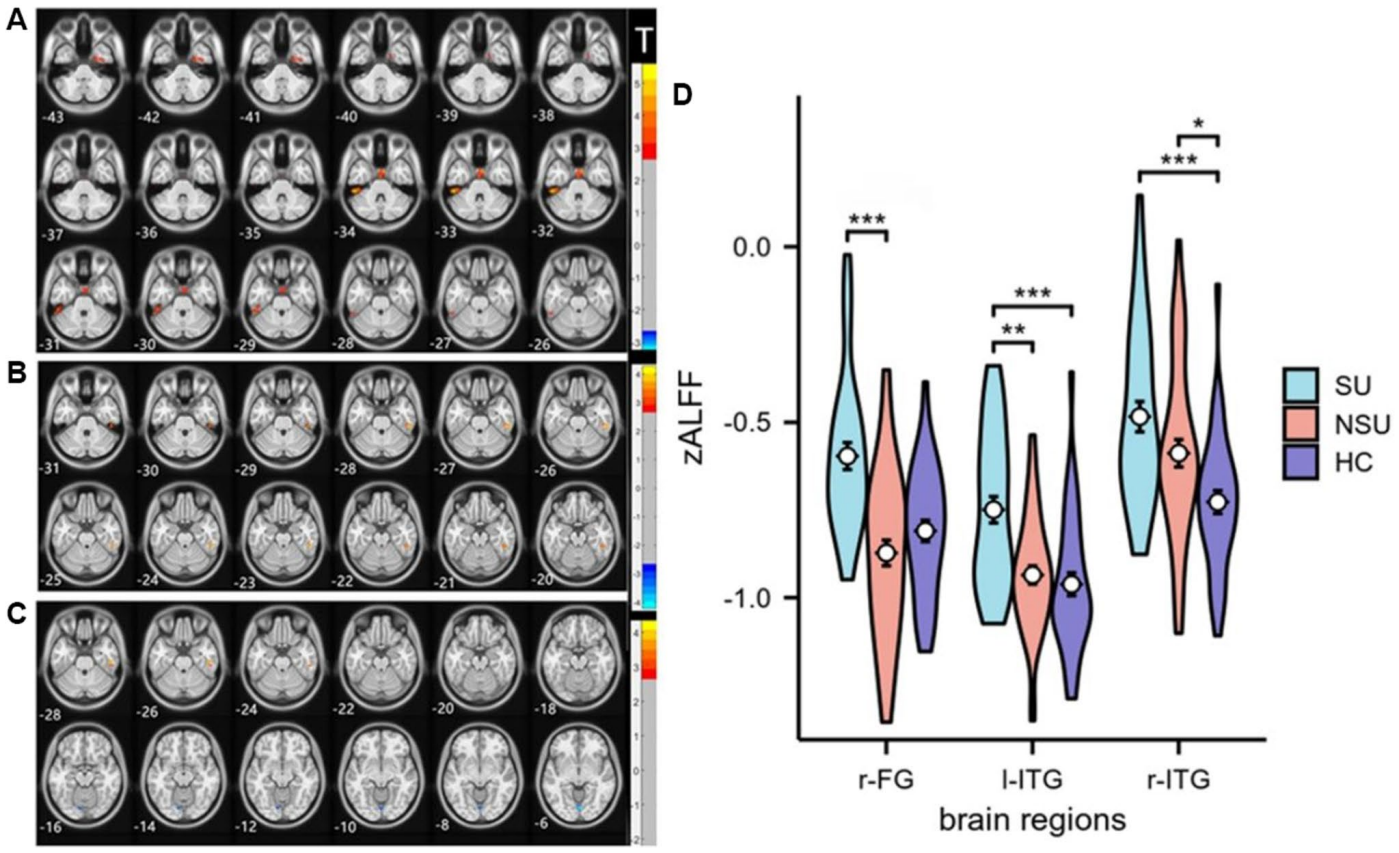
Statistical maps of *post hoc* t-tests for zALFF among the three groups: **(A)** SU group vs. NSU group; **(B)** SU group vs. HC group; and **(C)** NSU group vs. HC group. The red and blue colors represent increased and decreased zALFF, respectively. The color bar indicates the t-values based on *post hoc* analyses between the two groups. **(D)** ROI-wise comparisons of mean zALFF values across the SU, NSU, and HC groups. *denotes *p* < 0.05, **denotes *p* < 0.01, ***denotes *p* < 0.001. l-ITG: left inferior temporal gyrus, r-ITG: right inferior temporal gyrus, and r-FG: right fusiform gyrus.

As shown in Table 2, there were significant differences in zALFF values among the three groups in the left inferior temporal gyrus(l-ITG), right inferior temporal gyrus (r-ITG), and right fusiform gyrus(r-FG; GRF corrected, voxel-level *p* < 0.001, cluster-level *p* < 0.05). Post-hoc analysis revealed that the SU group had significantly increased zALFF in the l-ITG compared to both the NSU and HC groups. Additionally, the SU group exhibited higher zALFF in the r-FG compared to the NSU group, but not the HC group. Both the SU and NSU groups showed increased zALFF values in the r-ITG relative to the HC group (see Figure 1 and Table 2).

**Table 2:** Brain areas show zALFF differences among the SU, NSU, and HC groups.

Brain region (AAL)	Cluster size	MNI coordinates			Peak
	(voxels)	x	y	z	F/t
Three-group comparison					
l-ITG	53	−51	−36	−33	14.73
r-ITG	34	51	−27	−27	9.03
r-FG	45	36	−12	−45	13.33
SU > NSU					
l-ITG	24	−51	−36	−33	5.62
r-FG	15	36	−12	−15	4.37
SU > HC					
l-ITG	30	−60	−33	−27	4.12
r-ITG	28	54	−33	−27	4.29
NSU > HC					
r-ITG	12	48	−24	−27	4.38

Multiple comparisons were corrected using the GRF method, with the voxel level set at *p* < 0.001 and the cluster level set at *p* < 0.05. AAL: the Automated Anatomical Labeling atlas; MNI coordinates: coordinates in the Montreal Neurological Institute standard space. SU: Suicide Attempt; NSU: Non-Suicide Attempt; HC: healthy controls; l-ITG: left inferior temporal gyrus; r-ITG: right inferior temporal gyrus; r-FG: right fusiform gyrus.

### 3.3. Correlation between zALFF and clinical data

**Figure 2:**
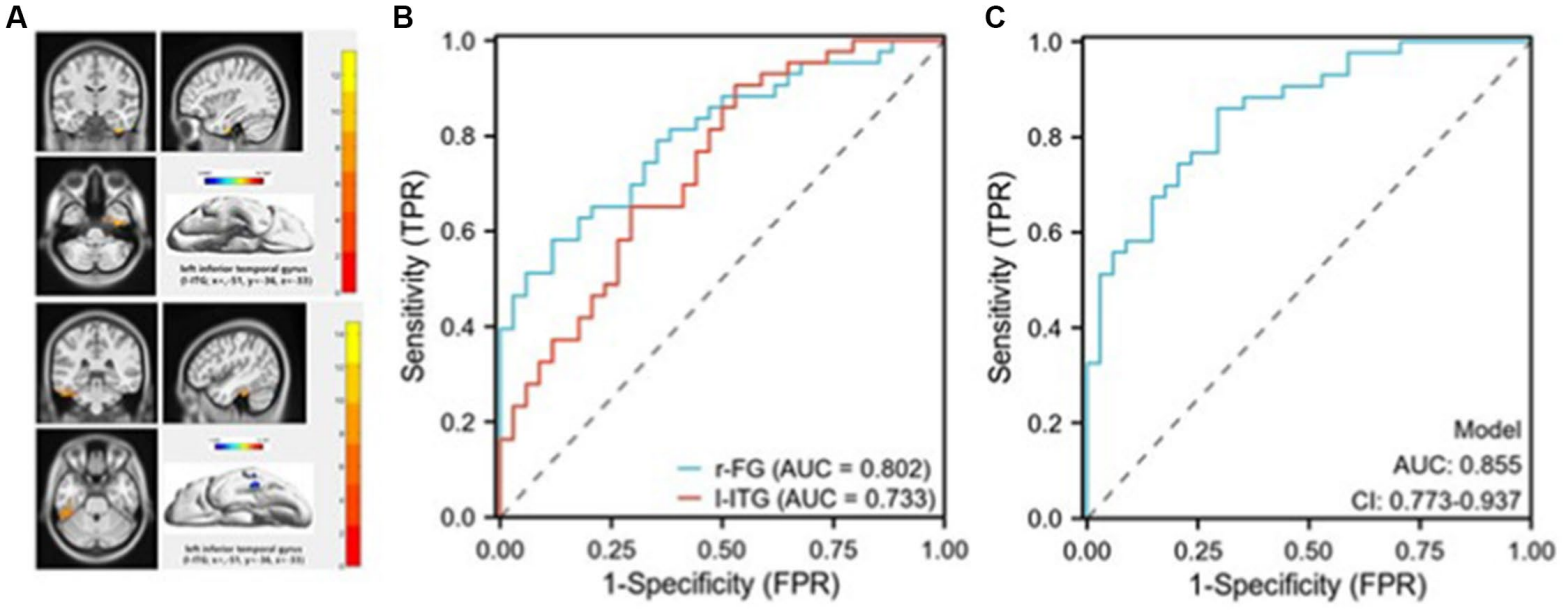
Receiver operating characteristic (ROC) curves for discriminating suicidal from non-suicidal depressed adolescents. **(A)** The masks (top image) represent the right fusiform gyrus (r-FG) region (coordinates: x = 36, y = −12, z = −15).The masks (bottom image) represent the left inferior temporal gyrus (l-ITG) region (coordinates: x = −51, y = −36, z = −33); **(B)** The ROC curve demonstrates the sensitivity and specificity of distinguishing between SU and NSU depressed adolescents by employing the mean zALFF values of the r-FG and l-ITG regions individually; **(C)** The ROC curve illustrates the sensitivity and specificity of differentiating between SU and NSU depressed adolescents using a combined model.

Partial correlation analysis, controlling for age and gender, revealed a significant negative correlation between DERS total scores and zALFF values in the left superior and middle frontal gyrus (l-SFG, l-MFG) in the SU group (*R* = −0.496, *p* = 0.003; *R* = −0.484, *p* = 0.005). No such relationship was observed in the NSU group (*R* = −0.18, *p* = 0.27; *R* = −0.16, *p* = 0.16). No significant correlations were found between other clinical indicators (HAMD scores, SSI scores) and zALFF values (see Figure 2). Using a less stringent GRF correction with a corrected voxel level of *p* < 0.005, we found a positive correlation between the number of suicide attempts and zALFF values in the left Precuneus in the SU group, and a negative correlation between SSI scores and zALFF values in the l-MFG in both the SU and NSU groups.

### 3.4. ROC analysis between SU and NSU groups

**Figure 3:**
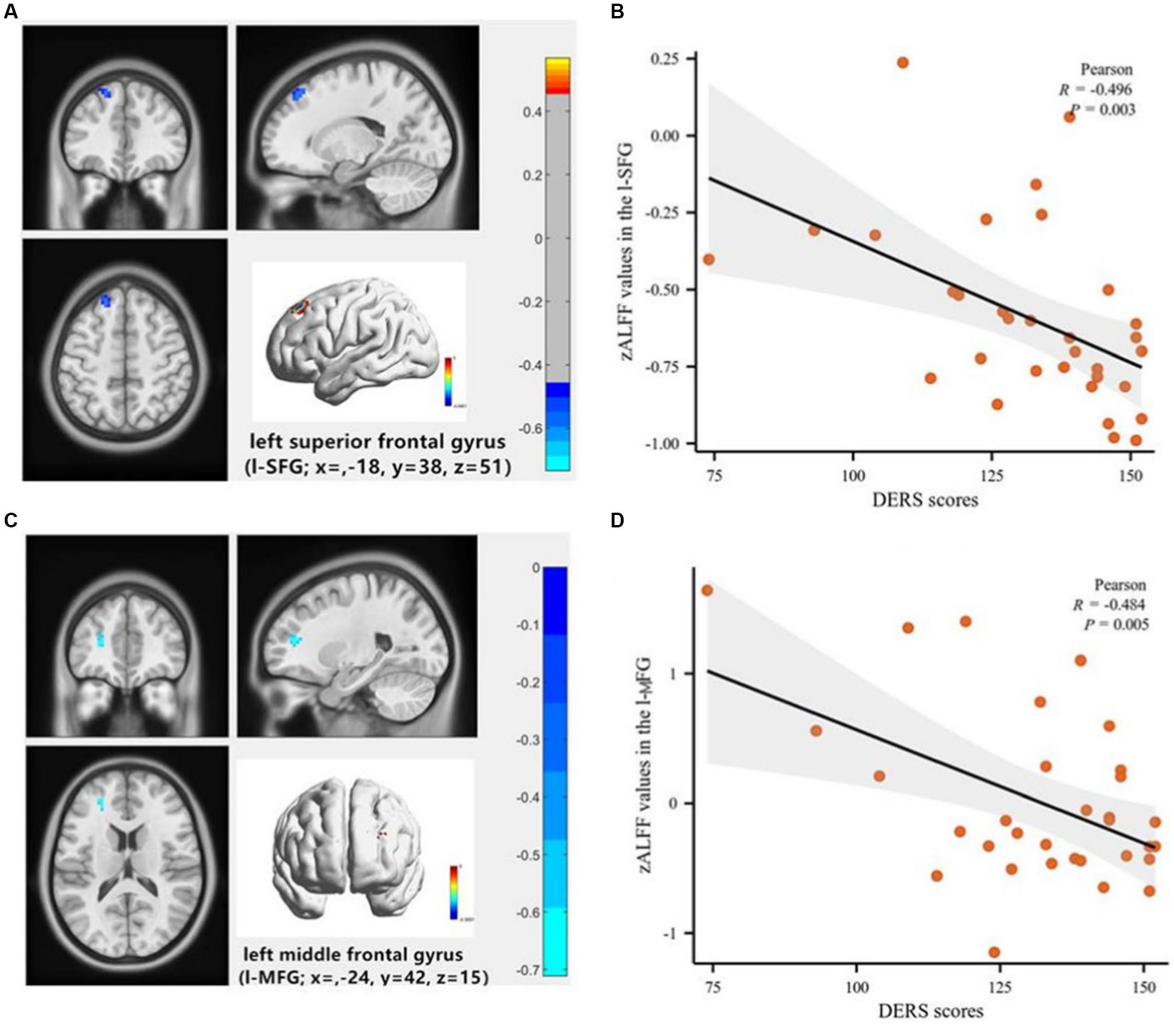
Voxel-wise correlation analysis of the association between Difficulty in Emotional Regulation Scale (DERS) scores and the zALFF maps of the whole brain in depressed adolescents with suicidal behavior. **(A)** The left superior frontal gyrus (l-SFG) (coordinates: x = −18, y = 38, z = 51), the color bar indicates the R-values based on Partial correlation analysis; **(B)** Scatter plot shows a negative correlation of DERS scores with zALFF values in the l-SFG (*R* = −0.496, *p* = 0.003). **(C)** The left middle frontal gyrus (l-MFG; coordinates: x = −24, y = 42, z = 15), the color bar indicates the R-values based on Partial correlation analysis; **(D)** The scatter plot shows a negative correlation of DERS scores with zALFF values in the l-MFG (*R* = −0.484, *p* = 0.005).

As shown in Figure 3, significant differences in zALFF values were observed in the r-FG and l-ITG regions between the SU and NSU groups, suggesting that these regions’ mean zALFF values could potentially serve as markers for distinguishing between suicidal and non-suicidal depression. ROC analysis revealed that the zALFF values in the r-FG region exhibited good discriminative capability, with an area under the curve (AUC) of 0.802, a sensitivity of 88.2%, and a specificity of 58.1%. Similarly, the zALFF values in the l-ITG region showed a fair AUC of 0.733, a sensitivity of 70.6%, and a specificity of 65.1% (see Figure 3). Furthermore, when combining the mean zALFF values of both the r-FG and l-ITG regions, relatively successful discrimination of SU patients from NSU patients was achieved, as evidenced by an AUC of 0.855(CI:0.773–0.973), a sensitivity of 86%, and a specificity of 71% (see Figure 3).

Finally, we conducted a sensitivity analysis to investigate medication effects. After excluding medicated patients, significant differences in zALFF values in the l-ITG and a negative correlation between DERS total scores and zALFF values in the left superior and middle frontal gyrus were still found. These results suggest that medication did not affect our main findings.

## 4. Discussion

The present study investigated the relationship between amplitude of low-frequency fluctuation (ALFF) abnormalities and suicidal attempts in depressed adolescents using resting-state functional magnetic resonance imaging (rs-fMRI). We found that both SU and NSU groups showed increased ALFF values in the inferior temporal gyrus (ITG) compared to the HC group and that the SU group had higher ALFF values in the left ITG (l-ITG) and right fusiform gyrus (r-FG) than the NSU group. Moreover, we found a negative correlation between ALFF values in the left frontal gyrus and scores of Difficulty in Emotional Regulation Scale (DERS) in the SU group, and a high accuracy of discriminating SU from NSU based on ALFF values from the r-FG and l-ITG. These findings of this study demonstrate that depressed adolescents with suicide attempts have distinct patterns of neural activity that reflect their social and emotion regulation impairments.

Our findings are partially in line with previous studies that reported abnormal ALFF values in the temporal and fusiform gyrus in depressed adolescents (7, 24, 25). For example, Cao et al. found that young depressed patients with suicidal behavior had increased baseline brain activity in the left middle temporal gyrus (l-MTG) compared to those without suicidal behavior (7). Huang et al. reported decreased functional connectivity between the l-FG and l-MTG in adolescents with major depressive disorder who engage in non-suicidal self-injury compared to healthy controls (24). Furthermore, previous research has shown a significant reduction in gray matter volume in the right temporal lobe of patients with a history of suicide attempts (26). In addition, the fusiform gyrus is involved in social cognition, such as decoding social cues and theory of mind (27). Our research, along with previous findings, suggests that abnormal brain activity and functional connectivity in specific brain regions, including the temporal and fusiform gyri, may play a role in the pathogenesis of depression and suicidal behavior in adolescents. However, there are also differences among studies, such as the specific brain regions identified and the populations studied (e.g., males vs. females) (25). The inferior temporal gyrus is involved in visual processing, memory, and emotion recognition (28), and the fusiform gyrus is associated with face perception, social cognition, and empathy (29, 30). These regions may be affected by the deficits in social and emotional processing that characterize depressed adolescents with suicide attempts (31, 32). For instance, previous studies have shown that depressed adolescents with suicide attempts have impaired facial emotion recognition (33, 34), reduced empathy, and altered social reward processing (35) compared to depressed adolescents without suicide attempts or healthy controls. Our findings suggest that aberrant neural activity in the ITG and fusiform gyrus may be related to past suicide attempts and infer a potential link to behavior in depressed adolescents.

According to the above literature review, the increased r-FG and l-ITG ALFF values in adolescent depression with suicide attempts may have several explanations. One possibility is heightened sensitivity to social cues and emotional expressions, leading to overactivation of these regions responsible for processing facial features and emotions (27, 29). Another explanation is dysfunction in the self-referential processing network, leading to an exaggerated negative self-image and distorted perception of reality. The r-FG and l-ITG regions may be part of this network, as they are also involved in self-face recognition and self-other distinction (29). A third possibility is a compensatory mechanism for impaired executive function. The r-FG and l-ITG regions may play a role in compensating for this impairment (36), as they are also involved in visual memory, attention, and cognitive control (28). More research is needed to confirm these hypotheses and understand the relationship between these brain regions and suicidal attempts.

In this study, we found a significant negative correlation between DERS scores and ALFF values in the l-SFG and l-MFG in depressed adolescents who had attempted suicide, but not in those who had not. This finding suggests that these brain regions may be involved in emotion regulation difficulties and past suicide attempts in this population. One possible explanation is that suicide attempts may reflect a more severe form of depression and emotion dysregulation, which may be associated with more pronounced alterations in brain function. Another possibility is that suicide attempts may have a causal effect on brain function. There is some evidence to support this idea. For example, studies have found that suicide attempters show prefrontal activation deficits during decision-making and inhibited increases in prefrontal blood flow compared to healthy controls (37). These findings suggest that suicide attempts may have an impact on brain function. Further longitudinal studies could help to clarify whether the observed changes in brain function are a consequence of suicide attempts or a risk factor for them.

The left superior frontal gyrus and the left middle frontal gyrus are part of the dorsolateral prefrontal cortex (DLPFC), which has been implicated in cognitive control, working memory, and executive functions (38, 39). Previous studies have reported reduced ALFF values in the DLPFC of depressed patients compared with healthy controls (40, 41), indicating impaired neural activity and connectivity in this region. Moreover, DLPFC dysfunction has been associated with suicidal ideation and attempts in depressed patients (42, 43). Our results are consistent with the existing literature and support the hypothesis that DLPFC dysfunction may contribute to emotion regulation difficulties and suicidal risk in depressed adolescents. Specifically, the left frontal lobe is an important component of both the cognitive control network (CCN) and the emotion regulation network (ERN), involved in higher cognitive functions such as working memory, decision-making, and executive function (44). as well as actively regulating negative emotional stimuli (45). Therefore, it may play an important role in emotion dysregulation in depressed adolescents.

Finally, the ROC analysis showed that the mean ALFF values in the r-FG and l-ITG region had a good discriminative capability between SU and NSU groups, indicating its potential as a biomarker for suicide attempts and infer a potential link to behavior in depressed adolescents. The l-ITG region also showed fair discriminative capability. Interestingly, when combining the mean ALFF values of both regions, relatively successful discrimination was achieved, suggesting that a combination of different biomarkers may provide a better prediction of suicide attempts and infer a potential suicide risk in depressed adolescents. Our research findings are consistent with some of the results in the literature but also have some differences (7, 25, 45). Unlike Cao et al. we did not find significant differences in the ALFF values in the l-SFG and l-MFG between the two groups, nor did this region have a good discriminative capability (7). This may be related to factors such as sample size, age range, severity of depression, etc. Our study and other studies have shown that ALFF can serve as a biomarker for suicidal and non-suicidal depressed adolescents, but the specific brain regions and more precise methods, such as machine learning methods, need further research.

Our findings have important clinical implications for the diagnosis and treatment of depressed adolescents with suicide attempts. First, our results suggest that ALFF may serve as a potential biomarker for identifying depressed adolescents who have past suicide attempts and infer a potential high risk of suicide. By combining ALFF values from the r-FG and l-ITG, we achieved a high accuracy in discriminating SU from NSU. This may help clinicians to screen for suicidal adolescents and provide timely intervention for this vulnerable population. Second, our results indicate that ALFF may reflect the underlying neural mechanisms of emotional dysregulation in depressed adolescents with suicide attempts. By targeting the brain regions with abnormal ALFF values, such as the l-SFG, cognitive behavioral therapy or neuromodulation techniques may improve emotional regulation skills and reduce suicidal ideation and behavior (46, 47). For example, previous studies have shown that cognitive behavioral therapy can enhance activation of the dorsolateral prefrontal cortex during emotion regulation tasks in depressed patients (48), and that transcranial magnetic stimulation can modulate ALFF values in the prefrontal cortex and improve depressive symptoms in suicidal individuals (49).

However, our study also has several limitations that should be acknowledged. First, our sample size was relatively small, which may limit the statistical power and generalizability of our findings. Second, our study was cross-sectional in nature, which precludes causal inference or assess changes in ALFF values over time. Additionally, we did not measure future suicide attempts so it is possible that the regional differences observed could be related to risk. However, without within-subject longitudinal data, it would be premature to assign any finding as ‘risk’. Third, it is important to note that our study did not control for potential confounding factors, such as non-suicidal self-injury, nor did we include a euthymic group with suicide attempts. This makes it difficult to discern whether the observed differences are specific to depression or represent trait differences between groups. These factors may influence ALFF values and potentially confound the observed association between ALFF and suicide attempts. Fourth, our study only used resting-state fMRI to measure ALFF values. Other imaging modalities, such as diffusion tensor imaging or functional connectivity analysis, may provide complementary information on the structural and functional integrity of the brain regions involved in depression and suicide. Finally, the exclusion of youth with psychiatric comorbidities and a history of substance or alcohol abuse/dependence in our study may limit the generalizability of our findings to a broader population of depressed adolescents who may have these comorbidities. Therefore, future research is needed to overcome these limitations and extend our findings. For example, larger and more diverse samples are needed to validate and replicate our results. Longitudinal studies are needed to examine the temporal dynamics and causal relationships of changes in ALFF values concerning depression severity and suicidal outcomes. Multimodal imaging studies are needed to explore the interaction and integration of different brain measures in depression and suicide. Intervention trials are needed to test whether modulating ALFF values can improve clinical symptoms and prevent suicidal behavior.

## 5. Conclusion

In conclusion, our findings suggest that depressed adolescents with suicidal behavior have distinct patterns of neural activity in the inferior temporal gyrus, fusiform gyrus, and frontal gyrus regions, which may contribute to their social and emotion regulation impairments and ultimately to suicidal behavior. Our study also highlights the potential of using mean ALFF values in these regions as markers for distinguishing between suicidal and non-suicidal depression. These findings may have implications for developing personalized treatment strategies for depressed adolescents with suicide attempts.

## Data availability statement

The raw data supporting the conclusions of this article will be made available by the authors, without undue reservation.

## Ethics statement

The studies involving human participants were reviewed and approved by The ethics committee of the Affiliated Hangzhou First People’s Hospital of Zhejiang University. Written informed consent to participate in this study was provided by the participants’ legal guardian/next of kin.

## Author contributions

CH designed the research, collected samples, analyzed data, and wrote the original manuscript. JieH, MW, and JL collected samples and analyzed data. JiaH and JX supervised data and conducted quality control. WJ supervised the research and revised the manuscript. YY supervised the research, provided funding, and gave some advice. All authors contributed to the article and approved the submitted version.

## Funding

This work was funded by National Natural Science Foundation of China (grant number: 81971277 and 82271570) and the medical and health research project of Zhejiang province (2023KY920).

## Conflict of interest

The authors declare that the research was conducted in the absence of any commercial or financial relationships that could be construed as a potential conflict of interest.

## Publisher’s note

All claims expressed in this article are solely those of the authors and do not necessarily represent those of their affiliated organizations, or those of the publisher, the editors and the reviewers. Any product that may be evaluated in this article, or claim that may be made by its manufacturer, is not guaranteed or endorsed by the publisher.
